# Training Doctoral Students to Advance Health Equity Through Policy Leadership: An Overview of the Health Policy Research Scholars Program

**DOI:** 10.1089/heq.2024.0159

**Published:** 2025-03-21

**Authors:** Keshia M. Pollack Porter, Attia Goheer, Jessica Harrington, Shannon Frattaroli

**Affiliations:** Department of Health Policy and Management, Johns Hopkins University Bloomberg School of Public Health, Baltimore, Maryland, USA.

**Keywords:** doctoral training, health policy, health equity, leadership, multidisciplinary

## Abstract

Health Policy Research Scholars (HPRS) is an innovative, equity-focused leadership program for doctoral students from marginalized backgrounds and identities. HPRS trains scholars from various disciplines to apply their research while engaging in policy to advance health equity. The HPRS logic model illustrates that training will lead to short-term changes including increased knowledge and skills; an interdisciplinary mindset; and increased sense of belonging, confidence, and self-awareness to advance health equity in the longterm. This article provides an overview of HPRS, including the logic model, curriculum, and implications for training doctoral students to become health equity leaders.

## Background

Achieving health equity is attainable through policy and systems reform, dismantling structural racism and power structures, and with scholarly leaders who are equipped with the knowledge, skills, and courage to embrace actions that support this vision. Few training opportunities exist for doctoral students who aspire to realize this vision. Most doctoral programs lack significant attention to the non-research skills necessary to prepare doctoral-trained scholars to advance health equity—understanding policy and systems change, leadership, effective cross-sector collaborations, and shifting power imbalances; partnering with communities; building coalitions; and engaging with policymakers, other decision-makers, and communities to enhance effective translation and dissemination of research findings.

The Health Policy Research Scholars (HPRS) program is part of the Robert Wood Johnson Foundation’s (RWJF) Leadership for Better Health portfolio.^1^ HPRS aims to build a community of doctoral students from marginalized backgrounds who will apply their research to advance health equity. This innovative national leadership program attracts doctoral students from various disciplines who are committed to conducting research, including with and informed by community, which informs policy to advance health equity so everyone has a fair and just opportunity to thrive.^2^ The 4-year primarily virtual training program, designed to complement full-time doctoral studies, welcomed its first cohort of 40 scholars in 2016. The final and ninth cohort under the current RWJF investment will begin in fall 2025. The program will end in 2029.

As of September 1, 2024, the HPRS community includes 150 active scholars and 177 alumni. Scholars and alumni from cohorts 1–8 represent institutions in 42 states and territories. One defining characteristic of HPRS is its inclusion of doctoral students from disciplines outside of public health, such as education, social work, and psychology; interdisciplinary training is critical to advance health equity. Current scholars and alumni represent 59 self-reported disciplines.

The purpose of this article is to describe the HPRS program, including the program’s logic model, to illustrate how HPRS will impact scholars in the short term and change systems, policies, and community in the long term. This article provides a program summary for doctoral programs to learn from and a foundation for future publications assessing the program’s impact.

### Program Overview and Current Program Status

The HPRS program was initially administered by a National Program Center (NPC) located at the Johns Hopkins Bloomberg School of Public Health (BSPH) from 2015 to 2017. In 2017, when the original director moved institutions, the NPC moved with the director. After a competitive selection process, the NPC returned to BSPH in 2019 under a new director. This paper focuses on the program’s current iteration (since September 2019) since the new NPC reimagined the curriculum by emphasizing leader and leadership development throughout the program.

HPRS is designed for full-time students in any research doctoral program from any U.S. institution, including institutions in the territories. Scholars apply for HPRS during their first year of their doctoral program and enter the program at the beginning of their second year. Applicants are evaluated based on written materials, including two letters of reference, one of which may be from a peer or community member. Semifinalists are identified for virtual interviews. Selection of the final cohort aims to include various disciplines; only 20–25% of selected applicants are from health or health-related fields. Policy expertise is not required for the program, but an appreciation for the role of policy change in advancing health equity is valued. Selection to the program is highly competitive, with an average acceptance rate of only 14%.

Scholars receive a $30,000 annual stipend from RWJF for up to 4 years to alleviate financial pressures associated with full-time doctoral study. They also receive access to financial and professional support from the NPC during and after their time in the program (i.e., as HPRS alumni). These supports include access to a competitive dissertation award, hardship funds for emergencies that might disrupt their doctoral studies and participation in HPRS (e.g., unexpected medical bills), and support from career mentors and leadership coaches.

Instruction based on the HPRS core competencies (Table 1) is primarily delivered virtually with monthly or semi-monthly sessions during the academic year, complementing scholars’ full-time programs. During years 1–3, scholars receive training in interdisciplinary approaches that bolsters their ability to identify policy-related questions and policy solutions to leading societal problems; advance health equity; promote policy change; engage in policy communications; and develop as leaders. They also practice policy-relevant skills through writing a policy memo and an op-ed, working in teams, and translating and communicating their research during visits with policymakers and advocacy organizations. Year 4 emphasizes the transition from student responsibilities to the workforce and includes professional development sessions. Throughout the program, scholars receive mentoring from leaders in the field and HPRS alumni and support navigating their doctoral programs at their home institutions.

**Table 1.** HPRS Core Competencies

Domains	Competency topics
Culture of Health and Health Equity	Awareness of the RWJF Culture of Health Framework and health equity
	Awareness of equity, diversity, inclusion, and anti-racism
	Awareness of health inequities and health determinants
	Awareness of health care and health systems
	Awareness of effective research methods and frameworks to advance a Culture of Health
Leadership	Awareness of effective leadership concepts and how to construct a unique leadership framework in support of implementing sustainable, equitable transformation
	Ability to work effectively with and within communities through partnership and coalition building while embracing diversity within communities and respecting differences
	Understanding of how to led relationally in dynamic and complex systems and environments through responsive and adaptive leadership
Health Policy	Knowledge of government structure and function to advance policies consistent with a Culture of Health
	Understanding of the roles of public and private sector stakeholders in policy strategies that advance a Culture of Health
	Awareness of the role of data, research, and evaluation in advancing policies consistent with a Culture of Health
	Appreciation for the role of implementation and how to advance it in the policy process
Communication	Ability to confidently advocate for oneself
	Ability to clearly and effectively communicate ideas and information both orally and in writing to individuals and groups
	Ability to apply methods of communication, including storytelling and data visualization, to reach audiences in new ways

HPRS, Health Policy Research Scholars; RWJF, Robert Wood Johnson Foundation.

A hallmark of HPRS is its emphasis on a leader and leadership development curriculum woven into each year’s course. Leader development focuses on intrapersonal growth to foster a leadership identity, while leadership development focuses on creating capacity to effectively work interpersonally and collaboratively.^3,4^ HPRS’ central concept of leadership as engaging in a process of social change is inspired by the Social Change Model of Leadership,^5^ and the Social Action and Leadership Transformation model.^6^ Year 1 emphasizes values, which includes assessing and naming one’s values and identifying how to cultivate and engage them through their doctoral studies and policy work. Year 2 builds on this knowledge and emphasizes identifying and nurturing one’s strengths with a leadership coach or through consistent reflection. With a foundation of values and strengths awareness, in year 3, the course is dedicated solely to leadership concepts. Scholars learn about the process of leading social change through course sessions focused on systems thinking, power, engaging with conflict, community organizing, and working collaboratively to create a shared narrative on a social issue. In year 4, scholars learn about self-leadership as they understand what it means to successfully navigate personal and professional transitions. Each scholar completes a leadership plan that includes articulating a leadership vision, philosophy, and several goals in support of their vision. All concepts can be practiced and applied throughout their doctoral experiences—whether teaching, researching, or engaging with the HPRS community or external communities, including at the scholars’ home institutions.

## Logic Model

**FIG. 1.**
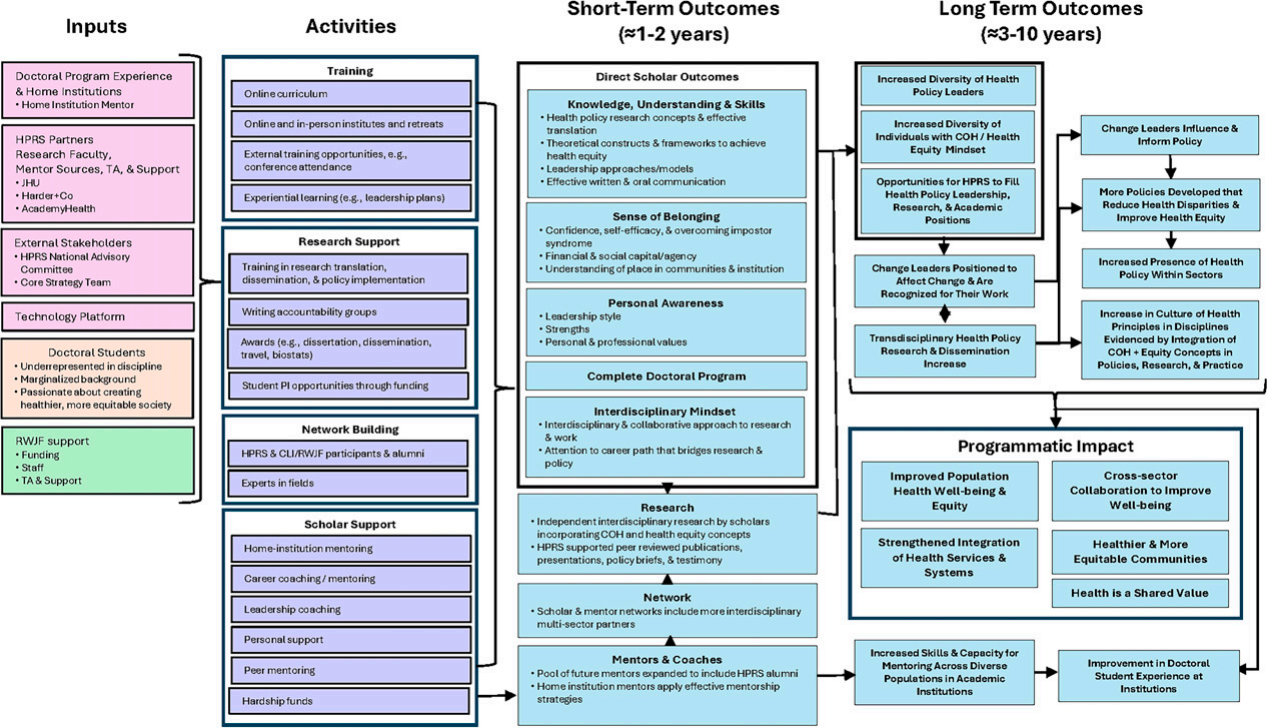
Health Policy Research Scholars Logic Model. Figure demonstrates the program components and theorized short-term and long-term impacts on the program participants and on advancing health equity.

After the NPC moved back to BSPH in 2019, the team developed a logic model that was built on what the prior NPC created (Fig. 1). The model illustrates RWJF’s initial emphasis on building a “Culture of Health” and the NPC’s vision for how building a community of scholars who are committed to research that will advance health equity through policy change and investing in that community will lead to societal impacts. Various supports facilitate the change, including didactic instruction in policy change and communication, as well as financial literacy; funds to attend conferences; dissertation awards; and access to hardship funds to buffer shocks that may disrupt and threaten doctoral studies. In addition to short-term outcomes related to change in knowledge, self-efficacy, and having an interdisciplinary mindset, long-term outcomes include advancing health equity practice and scholarship. Although not a goal of HPRS, it is noteworthy that 61% of all HPRS alumni as of this writing are in academic positions and poised to help transform academia by increasing the community of health equity researchers.

## Evaluating the HPRS Program

The impact evaluation for the HPRS program encompasses several data collection modalities and support from two evaluation partners. Annual data collection includes surveys, focus groups, and virtual interviews. Surveys fielded both by the HPRS external evaluation partner and a RWJF evaluation partner capture data on competency attainment, leadership style, identity, supports, social networks, policy work, and thought leadership activities, with additional reflection questions included on the exit survey for graduating scholars. Findings are shared with program participants on an *ad hoc* basis and inform HPRS program evolution. Evaluation data will be shared in future publications.

## Implications for Training to Advance Health Equity

HPRS is an example of innovative training that is equipping future doctoral degree recipients with the knowledge, skills, and confidence needed to advance health equity through evidence-informed policy change, while amplifying voices often excluded from these conversations. This program has several implications for training doctoral students as leaders for health equity that other institutions and programs could embrace. First, by emphasizing training in leader and leadership development, doctoral students’ leadership identities can flourish by identifying their values, strengths, and how their leadership guides their work. This type of training is typically lacking in doctoral programs and must be prioritized. Second, through promoting interdisciplinary and transdisciplinary instruction, students learn to explore the same issue from different disciplinary perspectives to collaborate and generate knowledge and solutions. For example, HPRS participants from various cohorts and disciplines collaborated on an article encouraging future transdisciplinary collaboration to dismantle structural racism.^7^ Doctoral studies are often siloed, and intentional efforts to connect students across disciplines can enhance their capacity to conduct research to address public health problems and advance equity. Third, doctoral students often find themselves alone in their home institutions because of their identity, values, interests, or some combination of these. HPRS provides a community that transcends geographic and institutional boundaries to build networks, friendships, and critical support for the doctoral journey. Doctoral programs can create equity-focused communities through short or long-term cohort-based programs, which may be especially important for scholars in programs with small cohorts of doctoral students interested in advancing health equity or from diverse backgrounds.

## Conclusions

HPRS is reimagining doctoral training to support institutional and societal change. This innovative program has impacts for advancing health equity through policy change research and practice.

## Abbreviations Used

BSPH: Johns Hopkins Bloomberg School of Public Health
HPRS: Health Policy Research Scholars
NPC: National Program Center
RWJF: Robert Wood Johnson Foundation
